# A Secreted Factor Coordinates Environmental Quality with *Bacillus* Development

**DOI:** 10.1371/journal.pone.0144168

**Published:** 2015-12-11

**Authors:** Qutaiba O. Ababneh, Amanda J. Tindall, Jennifer K. Herman

**Affiliations:** Department of Biochemistry and Biophysics, Texas A&M University, 2128 TAMU, College Station, Texas, United States of America; Loyola University Chicago, UNITED STATES

## Abstract

Entry into sporulation is governed by the master regulator Spo0A. Spo0A accumulates in its active form, Spo0A-P, as cells enter stationary phase. Prior reports have shown that the acute induction of constitutively active Spo0A during exponential growth does not result in sporulation. However, a subsequent study also found that a gradual increase in Spo0A-P, mediated through artificial expression of the kinase, KinA, during exponential growth, is sufficient to trigger sporulation. We report here that sporulation via KinA induction depends on the presence of an extracellular factor or factors (FacX) that only accumulates to active levels during post-exponential growth. FacX is retained by dialysis with a cutoff smaller than 500 Dalton, can be concentrated, and is susceptible to proteinase K digestion, similar to described quorum-sensing peptides shown to be involved in promoting sporulation. However, unlike previously characterized peptides, FacX activity does not require the Opp or App oligopeptide transporter systems. In addition, FacX activity does not depend on SigH, Spo0A, or ComX. Importantly, we find that in the presence of FacX, *B*. *subtilis* can be induced to sporulate following the artificial induction of constitutively active Spo0A. These results indicate that there is no formal requirement for gradual Spo0A-P accumulation and instead support the idea that sporulation requires both sufficient levels of active Spo0A and at least one other signal or condition.

## Introduction

A major challenge in developmental biology is to uncover the signals that stimulate differentiation. Bacteria use cell-cell signaling to receive a variety of spatial, temporal and environmental cues that help them regulate and coordinate the requisite morphological and physiological changes needed for differentiation [1]. Quorum sensing is one form of cell-cell signaling that enables bacteria to share information about the population density and to respond by reprogramming gene expression [2]. In quorum sensing, bacteria use diffusible molecules, such as acyl-homoserine lactones, that increase in concentration with cell density. When a critical threshold of signal accumulates, a population-based community behavior, such as the production of bioluminescence, is induced [2,3]. The growing list of bacterial processes regulated by quorum sensing includes extracellular enzyme secretion [4], antibiotic production [5,6], virulence [2], competence for DNA uptake [7,8], biofilm formation [9,10], and sporulation [11,12].

*Bacillus subtilis* is a Gram positive organism capable of differentiating into multiple cell types, including heat and desiccation resistant spores [13]. Spore formation can be induced through nutrient exhaustion [14] or through conditions that cause a rapid fall in cellular GTP levels [15,16] However, Grossman and Losick observed that sudden drops in GTP levels are insufficient to trigger efficient sporulation when cell densities are very low [11]. This observation ultimately lead to the discovery of oligopeptide-mediated quorum-sensing in *B*. *subtilis* [11].

The oligopeptide signals of *B*. *subtilis* are synthesized ribosomally as pro-peptides, secreted outside the cell, processed into the mature oligopeptide forms, and then transported back into the cell by the oligopeptide uptake systems Opp and App [17]. Once internalized, the processed peptides promote the phosphorylation of the global response regulator Spo0A [18]. During transition and stationary phase, Spo0A levels rise and the active form, Spo0A-P, accumulates [19,20]. At lower levels, Spo0A-P activates and represses genes involved in growth phase adaptation nutrient scavenging and competence [20,21]. At higher levels of Spo0A-P, the developmental pathway of sporulation is initiated [20]. The signaling network regulating Spo0A’s phosphorylation state is complex [22]. Several sensor kinases, including KinA, promote Spo0A phosphorylation [23,24]. Conversely, several phosphatases antagonize Spo0A phosphorylation both directly and indirectly [24]. The characterized quorum sensing oligopeptides of *B*. *subtilis* inhibit the activity of the phosphatases, promoting development by shifting Spo0A toward its phosphorylated form [25]. Since, the known quorum-sensing peptides of *B*. *subtilis* act as modulators of Spo0A-P levels, these results suggest that the sporulation pathway is primarily a function of Spo0A-P levels, and that the contribution of cell density and/or growth phase is indirect.

In order to probe this hypothesis directly, Ireton and colleagues isolated a constitutively active alelle of *spo0A* called *sad67* and placed it under the control of an inducible promoter (P_*spac*_) [26]. Although induction of the *SAD67* variant resulted in expression of early sporulation genes [20,26–28], it was not sufficient to induce efficient sporulation under nutrient replete conditions unless the cells were also treated with decoyinine to cause a rapid drop in GTP levels or allowed to enter stationary phase [26]. These results suggested that there are at least two requirements for efficient sporulation: sufficient levels of active Spo0A and a signal indicating deteriorating environmental conditions.

In a subsequent study, Fujita and Losick found that *B*. *subtilis* could be triggered to sporulate efficiently in rich media (specifically CH) if the Spo0A-P levels were elevated gradually; this gradual accumulation could be achieved by artificially expressing KinA [29], one of several kinases that donate phosphoryl groups to the Spo0A phosphorelay. The authors concluded that Spo0A-P was both necessary and sufficient to promote sporulation during exponential growth in rich media [29]. Moreover, the authors suggested that nutrient-dependent signals, such as GTP levels, likely act to promote sporulation only indirectly, by feeding into the Spo0A phosphorelay.

In the present study, we find that *B*. *subtilis* cells always maintained at exponential phase cell densities (OD_600_ of < 0.7 in CH medium) are unable to sporulate through KinA-dependent induction. Instead, we find that sporulation requires both KinA induction and the presence of sufficient levels of at least one extracellular signal, which we call Factor X (FacX). FacX is retained by dialysis with a cutoff smaller than 500 Dalton, is heat stable, and is sensitive to proteinase K, consistent with previously characterized quorum-sensing peptide-based signals shown to be involved in sporulation. However, FacX activity is not dependent on Spo0A, SigH, or ComX [30], and does not require the Opp and App oligopeptide transporters [31–33]. Finally, spiking cultures with concentrated media containing FacX is sufficient to induce sporulation at low cell densities when a constitutively active variant of Spo0A (Spo0A*) is expressed, suggesting that there is no formal requirement for gradual Spo0A-P accumulation in sporulation induction. Moreover, these results indicate that FacX does not act through the phosphorelay pathway, as Spo0A* acts independently of phosphorylation. In sum, our results indicate that Spo0A-P is necessary, but not sufficient to trigger sporulation. Instead, efficient sporulation requires both sufficient levels of active Spo0A-P and at least one other signal.

## Materials and Methods

### General methods

The *B*. *subtilis* strains used in this study are listed in Table 1. All strains were streaked on LB plates (10 g/L tryptone, 5 g/L yeast extract, 5 g/L NaCl, and 1.5% Bacto agar). Cultures were grown aerobically at 37°C in liquid Casein Hydrolysate (CH) medium, a nutrient rich medium [34]. *Escherichia coli* DH5α and TG-1 were used for isolation of plasmid DNA and were grown in LB medium. Competent *B*. *subtilis* cells were generated as described previously [35]. When needed, antibiotics were added to the growth media at the following concentrations: 100 μg/ml spectinomycin, 7.5 μg/ml chloramphenicol, 5 μg/ml kanamycin, and 1 μg/ml erythromycin plus 25 μg/ml lincomycin (mls), for *B*. *subtilis* strains; and 100 μg/ml ampicillin, for *E*. *coli* strains.

**Table 1:** Strains and oligos used in this study.

Strain	Relevant Genotype	Reference
PY79		[36]
BQA019	*spo0H*::*cat in B*. *subtilis 3610*	[37]
BKE31700	*comX*::*erm*	This work
**PY79**		
BDR2051/EH269	Δ*spo0A*::*kan*	David Z. Rudner
BQA023	Δ*sigH*::*cat*	This work
BQA061	Δ*oppABCDF*::*spec*	This work
BQA119	Δ*oppABCDF*::*spec*, *kinAΩP*_*hy-spank*_ *-kinA (cat)*	This work
BQA121	Δ*oppABCDF*::*spec*, *kinAΩP*_*hy-spank*_ *-kinA (cat)*, Δ*appA*::*kan*	This work
BQA122	*comX*::*erm*	This work
MF1913	*kinAΩP*_*hy-spank*_ *-kinA (cat)*	[29]
MF2146	*spo0AΩ*P_*hy-spank*_-spo0A* *(spec)* (*sad67)* allele)	[29]
**oligo**	**5’ to 3’**	
oAS078	GGATCCCAGCGAACCATTTGA	
oAS079	GTCGACAAATTCCTCGTAGGC	
oJH217	TTACTCGAGCTCTTAACTGATCTGCTGCTT	
oJH218	AAGCAGCAGATCAGTTAAGAGCTCGAGTAA	
oJH219	CCGAGCCGAATTCTTTCTCTA	
oJH220	GGATCGGCCGGCTGGATTCAA	
oQA170	TCAAAGAAAAGTCGGTGATGT	
oQA171	CCTATCACCTCAAATGGTTCGCTGGGATCCTAGATATTCCCCCTCTTGAAATGT	
oQA172	GTCCCGAGCGCCTACGAGGAATTTGTCGACATTTCCCTTAAAGGGGAG	
oQA173	TCGCGTAAAGGATGTCTGCAA	

### Strains and plasmid construction

Unless indicated otherwise, PY79 genomic DNA was used as template in the PCR reactions.

BQA023 (Δ*sigH*::*cat*). To move Δ*sigH*::*cat* into a PY79 background (isogenic to the other strains used in this study) genomic DNA from BQA019 (Δ*sigH*::*cat* in *B*. *subtilis* 3610) was transformed into PY79, and selected for on LB supplemented with chloramphenicol.

BQA061 (Δ*oppABCDF*::*spec)*. To generate a deletion of the *opp* operon, a region beginning at the second codon in *oppA* until the last three codons in *oppF* was replaced with a spectinomycin cassette as follows: PCR product from oJH219 and oJH220 was cut with EcoRI-BamHI and ligated into the spectinomycin resistant allelic exchange vector pKM079 (David Rudner) cut with EcoRI-BamHI to generate pJH041. PCR product amplified using oJH217 and oJH218 and digested with EagI-SalI was ligated into pJH041 digested with EagI-SalI to generate pJH042. The resulting pJH042 plasmid was linearized with ScaI and introduced into *B*. *subtilis* PY79 by transformation to create BQA061. Deletion of the operon was confirmed by PCR.

BQA119 (Δ*oppABCDF*::*spec*, *kinAΩP*_*hy-spank*_ *-kinA (cat)*). MF1913 was transformed with genomic DNA from BQA061 (Δ*oppABCDF*::*spec)*, followed by selection on LB spectinomycin plates. Deletion of the operon was confirmed by PCR.

BQA121 (Δ*oppABCDF*::*spec*, *kinAΩP*_*hy-spank*_ *-kinA (cat)*, Δ*appA (kan)*). The appA gene deletion was generated by transformation of a enzymatic assembly reaction. To generate the enzymatic assembly, three PCR products were amplified: an “UP” region that encompassed ~1000 nt region upstream of *appA* and ended immediately before the start codon was amplified using primer pair oQA170 and oQA171; a “DOWN” region that began immediately after the stop codon and encompassed ~1000 downstream of *appA* was PCR amplified with primer pair oQA172 and oQA173; a kanamycin resistance cassette with overhangs that matched oQA171 and oQA172 was generated PCR amplified from pXW114 (gift from David Rudner, Harvard Medical School) using primer pair oAS078 and oAS079. The three products were combined in a single enzymatic assembly reaction [38] for 60 min. The contents of the reaction (20 ul total volume) were used in a PY79 transformation, and transformants were selected for on LB plates supplemented with kanamycin. Deletion of *appA* was confirmed by PCR.

### Microscopy

Culture samples (1 ml) were collected and pelleted by centrifugation at room temperature at 6,010 x g in a tabletop microfuge. The supernatants were removed using aspiration and resuspended in ~10 μl PBS containing the indicated dyes at the following final concentrations: 2.0 μg/ml DAPI DNA stain (Molecular Probes); 0.02 mM TMA-DPH (Life Technologies) or 3.0 μg/ml FM4-64 (Life Technologies). After resuspension, samples were mounted on glass slides with polylysine-treated coverslips. Exposure times were generally 1 sec. Images were collected with a Nikon Ti-E microscope equipped with a CFI Plan Apo lambda DM 100X objective, and Prior Scientific Lumen 200 Illumination system, C-FL UV-2E/C DAPI, and C-FL Texas Red HC HISN Zero Shift filter cubes, and a CoolSNAP HQ2 monochrome camera. All obtained images were captured with NIS Elements Advanced Research (version 4.10), and processed with ImageJ64 [39].

### Conditioned CH media preparation

Single colonies were used to inoculate 3 ml CH cultures. Fifty μl of starter culture was then used to inoculate 500 ml CH media in a 2.5 L flask. The culture was grown in a shaking waterbath at 300 rpm, 37°C to an OD_600_ nm between 1.3 and 1.5. We found that conditioned media collected from cells grown to an OD_600_ value of 1.2 supported less robust sporulation. Cells were removed by centrifugation at room temperature, 17,700 x g for 10 min and the conditioned media was filter sterilized by passing it through a 0.45 μm filter. All conditioned media was tested for activity using the sporulation assay (described below) and stored at 4°C until needed. The activity was found to be stable for at least one month at 4°C. When indicated, the conditioned media was concentrated using a rotovap in a 30°C waterbath until reaching a 50X or greater concentration.

### Heat treatment, proteinase K treatment and dialysis of conditioned media

Heat treatment of conditioned CH media was carried out by transferring the conditioned media to a glass tube and submerging the glass tube in a bath of boiling water for 15 min. The heat-treated media was cooled before testing in the sporulation assay. To treat the conditioned CH media with protease, 30 mg of proteinase K conjugated to agarose beads (Sigma) was hydrated in 3 ml ddH_2_0 for 20 min at room temperature, and then centrifuged at 1,292 x g for 2 min. After removing the supernatant, the beads were washed in 3 ml ddH_2_0, and pelleted by centrifuging at 1,292 x g for 2 min. The wash was repeated two more times. After the final centrifugation, the beads were resuspended in 10 ml conditioned media and placed at 37°C with gentle agitation for 1 hr. After incubation, the sample was centrifuged at 1,292 x g for 2 min. The supernatant was passed through a 0.2 μm filter to remove any residual beads and stored at 4°C overnight before testing in the sporulation assay. Conditioned CH media was dialyzed against CH media (1 part conditioned media to 200 part fresh CH) overnight at 4°C. Following exchange to fresh CH, dialysis was carried out for 8–10 hrs at 4°C. As a final dialysis step, the CH was exchanged for fresh CH again and the dialysis was again carried out overnight. The dialysate present inside the tubing was collected, sterilized with a 0.45 filter, and stored at 4°C until use. All dialysis tubing was composed of cellulose ester. The following dialysis tubing was utilized: 0.1–0.5 kDa (Spectrum laboratories), 1.0 kDa (Spectrum laboratories), 3.5 kDa (Snakeskin, Thermo Scientific).

### Sporulation assay

A single colony of the strain of interest (this varied by experiment, see results) was used to inoculate 5 ml of CH medium, and the cells were grown at 37°C to an OD_600_ of between 0.2 and 0.6. This culture was used to inoculate 25 ml of CH medium in a 250 ml baffled flask, and cells were grown in a shaking waterbath at 300 rpm, 37°C to an OD_600_ of between 0.4 and 0.6 to ensure the cells were in exponential growth. At this point, the assay was performed on one of two scales with indistinguishable results: either 250 ml baffled flasks, with volumes of media up to 25 ml, or in 18 mm glass test tubes with volumes up to 3 ml. We did not test other volumes. For the larger scale assay, 2 ml of the exponentially growing culture was added to 23 ml of pre-warmed conditioned media in a 250 ml baffled flask, and the culture is placed in a shaking waterbath set to 250 to 300 rpm. To induce KinA expression, 20 μM of IPTG (final concentration) was added, as previously described [29]. In the smaller scale assay, 200 μl of the exponentially growing culture was added to 2.3 ml of conditioned CH in 18-mm glass tubes and placed in a shaking waterbath set at 300 rpm, 37°C. To induce KinA expression, 20 μM of IPTG (final concentration) was added to the 2.5 ml in the sporulation assay tube. Culture tubes were incubated in a shaking waterbath at 300 rpm, 37°C for 2 hrs. The OD_600_ values at the end of the incubation period generally fell between 0.2 and 0.4 (this varied by batch of conditioned media), and were always below an OD_600_ of 0.6. One ml of sample was used to check assay for the onset of sporulation by microscopic analysis as described above. For the P_hy-spank_-Spo0A* (MF2146) induction experiment in conditioned media, the sporulation assay was performed in 250 ml baffled flasks (as described above) and cells was induced with either 200 μM IPTG (as previously described [29]) or 500 μM IPTG. Cells grown at both concentrations of inducer initiated sporulation (as judged by the presence of forespores), however the induction of sporulation with 500 μM IPTG appeared slightly more robust.

## Results

### Sporulation through artificial KinA induction requires exit from exponential growth

**Fig 1:**
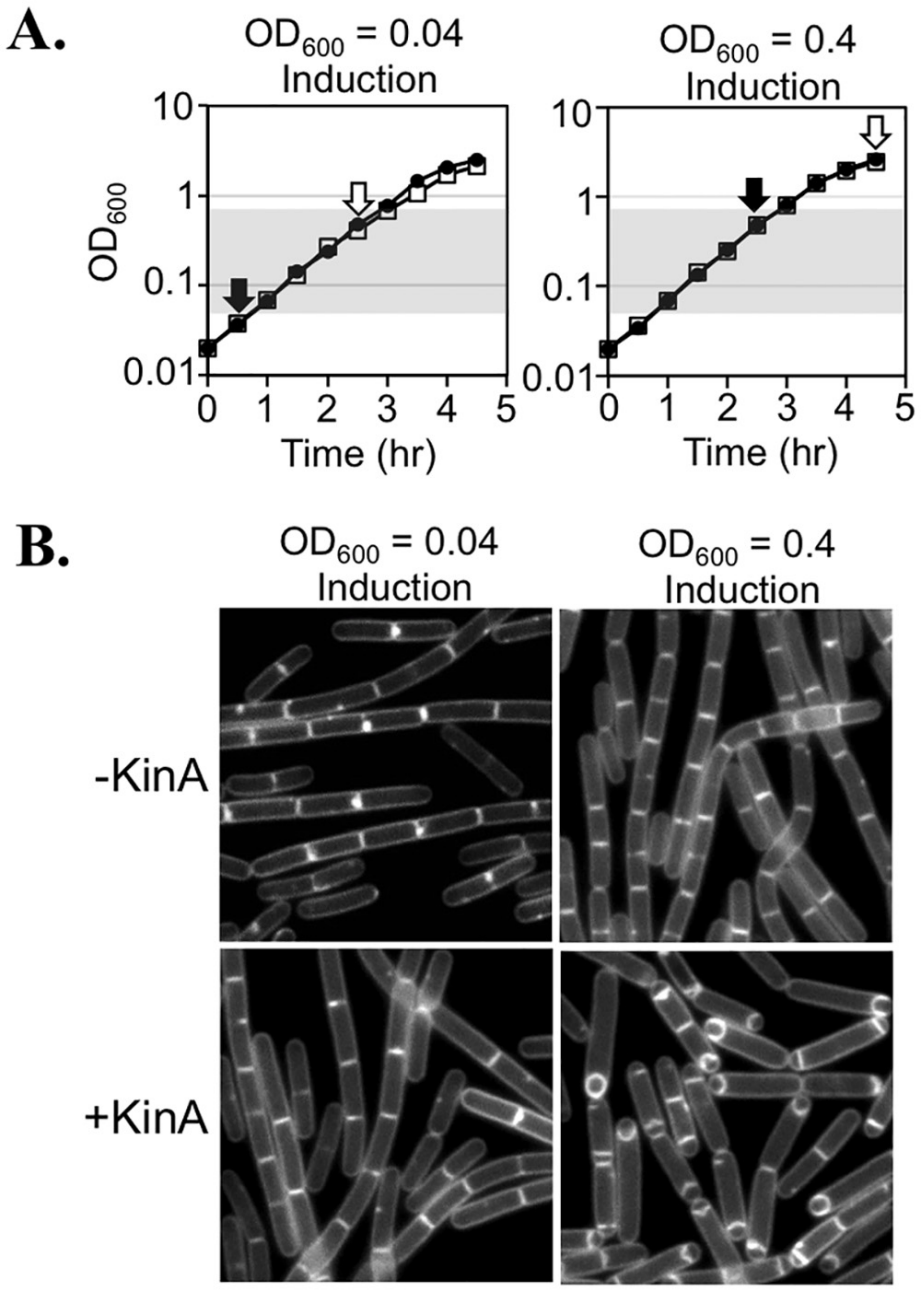
KinA-dependent sporulation requires exit from exponential growth. (A). A strain harboring P_*hy-spank*_-*kinA* (MF1913) was grown to mid-exponential phase, back-diluted to an OD_600_ = 0.02, and split into four flasks. At the cell densities indicated by the black arrows, KinA expression was induced by the addition of 20 μM IPTG. On the growth curve, closed circles correspond to uninduced samples, while open squares correspond to induced samples. Shading indicates the region of exponential growth observed in the uninduced samples. A reduction in growth rate of culture induced at OD_600_ = 0.04 (left, open squares) was observed beginning between 1.5 and 2 hr post-induction. Cells were grown 2 hrs before image capture (image capture cell densities are indicated by white arrows). (B) Images of P_*hy-spank*_-*kinA* (MF1913) taken at the timepoints indicated by the white arrows in (A). Membranes are stained with TMA.

*B*. *subtilis* can be induced to sporulate in CH (a rich medium) when cells are engineered to artificially express KinA during exponential growth (see introduction). In the *B*. *subtilis* strain utilized in this study (PY79), exponential phase is defined as cultures growing at a linear rate (log scale) in liquid CH medium at 37°C (OD_600_ values of 0.04 to 0.7, Fig 1A, shaded region of growth curve). While investigating the effects of KinA induction on DNA replication during vegetative growth in CH medium, we observed that KinA-induced cells that were maintained in exponential phase did not manifest morphological signs of sporulation (loss of cell chaining, polar septa, axial filament formation, and engulfment), even after two hrs of growth (Fig 1B). Less than 2% of cells (n = 508) exhibited polar septation or forespores. Instead, the cells appeared similar to the uninduced control, in which less than 1% of cells (n = 564) exhibited polar septation or forespores (Fig 1B). In contrast, if KinA-induced cells were allowed to exit exponential growth, 75% of cells (n = 744) exhibited polar septa and forespores (Fig 1B), morphological changes characteristic of sporulation initiation. The ability of the cells to initiate sporulation required both KinA induction and exit from exponential growth, as less than 1% of cells in the uninduced control possessed polar septa or forespores (Fig 1B). This experiment was repeated in three independent biological replicates, each time with similar results. We conclude that robust sporulation initiation in CH media through artificial KinA induction requires that cells achieve OD_600_ values of higher than 0.7 (the highest value exhibiting exponential growth for which we assayed for sporulation phenotypes in the present study).

### Media from post-exponential growth contains a factor that promotes sporulation via KinA induction

**Fig 2:**
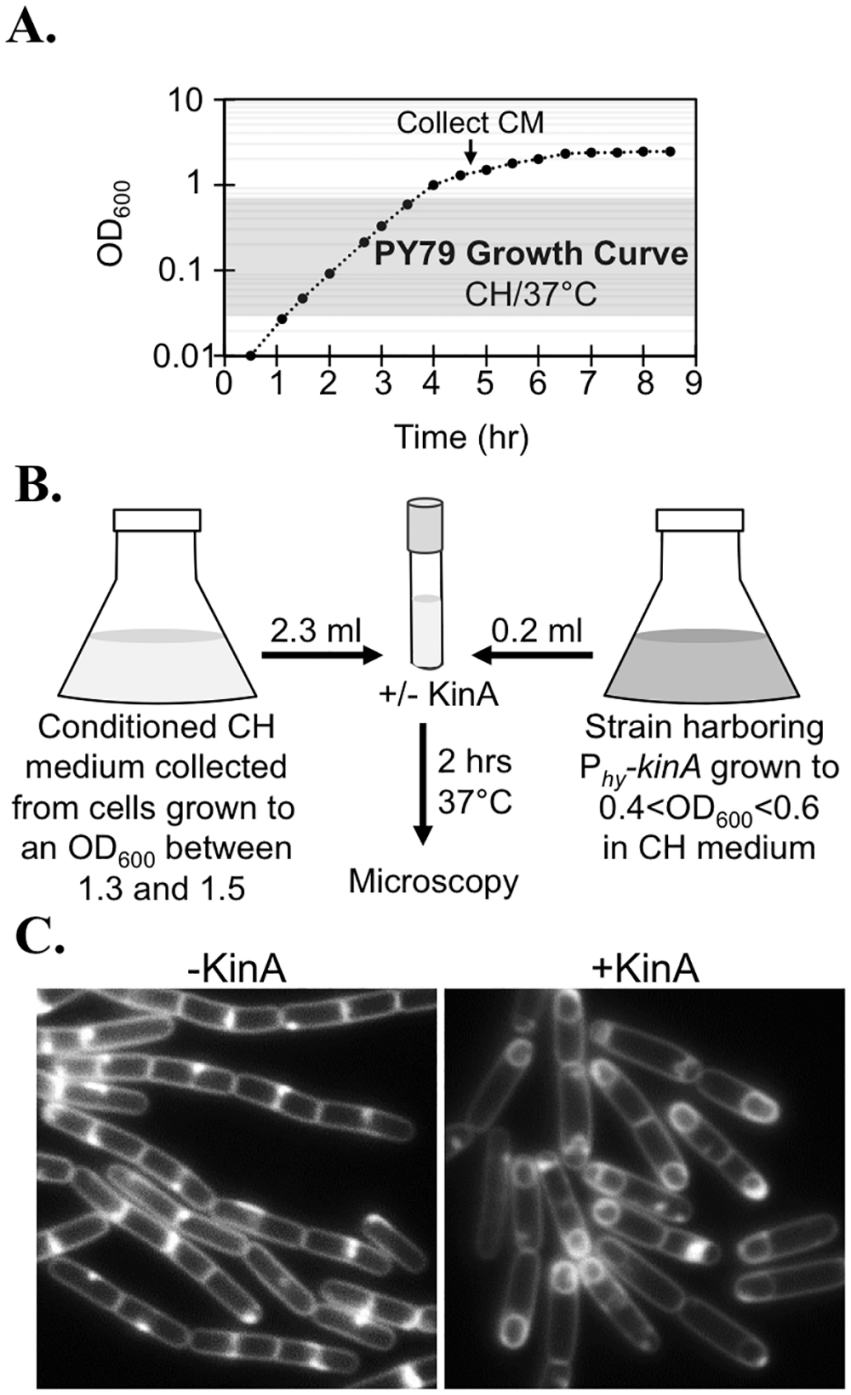
Media from post-exponential growth contains a protein-based factor that promotes sporulation via KinA induction. (A) A typical PY79 growth curve in CH medium at 37°. The arrow indicates region in the growth curve (OD_600_ between 1.3 and 1.5) where the conditioned media (CM) was collected. The shaded region indicates the region of the growth curve in which exponential growth occurs. (B) Schematic representation of the KinA-dependent sporulation assay used throughout this study. (C) A strain harboring P_*hy-spank*_-*kinA* (MF1913) was grown as described in (A) with conditioned media collected from PY79. KinA was induced with IPTG (20 μM final concentration). Membranes were stained with TMA.

*B*. *subtilis* is known to produce extracellular oligopeptides that stimulate sporulation in a cell density dependent manner [25], so we hypothesized that KinA-dependent sporulation might require the accumulation of an extracellular factor that only accumulated to active levels in cultures grown to post-exponential phase in CH media. To test this idea, we grew *B*. *subtilis* in liquid CH media until the culture reached an OD_600_ of between 1.3–1.5, then removed the cells by centrifugation and filtration to generate “conditioned media” (Fig 2A). We then inoculated the conditioned media with a small volume of exponentially growing cells harboring P_*hy-spank*_ *-kinA*. If no inducer (IPTG) was added, the cells continued to grow primarily as chained cells that lacked polar septa (Fig 2B). These results indicate that the conditioned media itself is not sufficient to induce sporulation in the timecourse of this experiment. In contrast, when KinA was induced in cells growing in the conditioned media, robust sporulation initiation was observed after 2 hr (Fig 2B). This experiment was repeated over ten times with independent biological replicates and independent batches of conditioned media with similar results.

**Fig 3:**
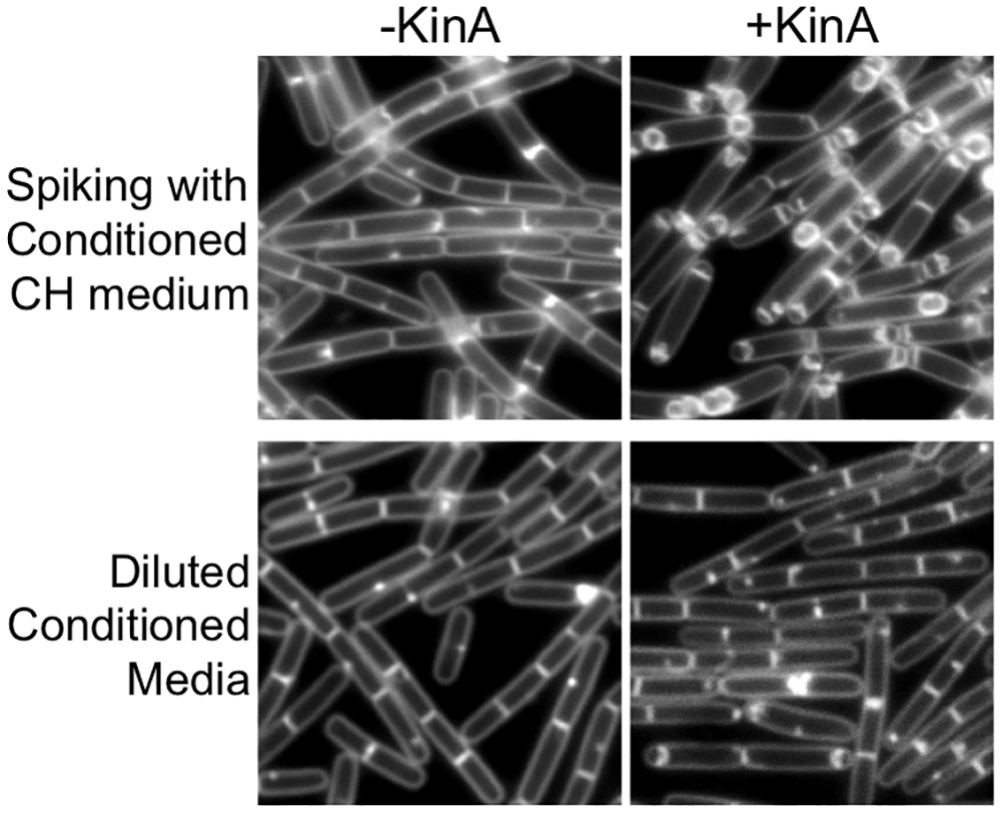
KinA-dependent sporulation requires a threshold level of FacX. MF1913 (P_*hy-spank*_-*kinA*) was grown to exponential phase and 0.2 ml of culture was used to inoculate either fresh CH spiked with 37.5 ul of 67X concentrated conditioned media (1X final concentration)(top) or diluted conditioned media (3 parts conditioned CH to 1 part fresh CH) (bottom). When indicated, *kinA* expression was induced with IPTG (20 μM). Cells were grown for 2 hrs (top panels) or 2.5 hrs (bottom panels) at 37°C before image capture. Membranes were stained with TMA.

We next determined if the sporulation-inducing activity could be concentrated or diluted, which would suggest the presence rather than the absence of a factor that made sporulation permissive through artificial KinA induction. When cells growing in fresh CH medium were spiked with concentrated conditioned media at a 1X concentration, polar septation and engulfment were observed most cells (Fig 3). However, diluting the conditioned media by even 25% (3 parts conditioned media mixed to 1 part fresh CH) reduced the percentage of cells exhibiting polar septation or engulfment down to 7% (n = 819)(Fig 3). These results suggest that the sporulation-stimulating activity is concentration-dependent, and that a critical threshold of the activity is required to stimulate KinA-dependent sporulation. We conclude that in addition to the accumulation of Spo0A-P (in this case via artificial KinA induction), cells require the presence of at least one additional factor for efficient sporulation at low cell densities. We refer to this factor (or factors) as FacX.

### FacX is sensitive to protease and retained by dialysis membranes smaller than 1 kDa

**Fig 4:**
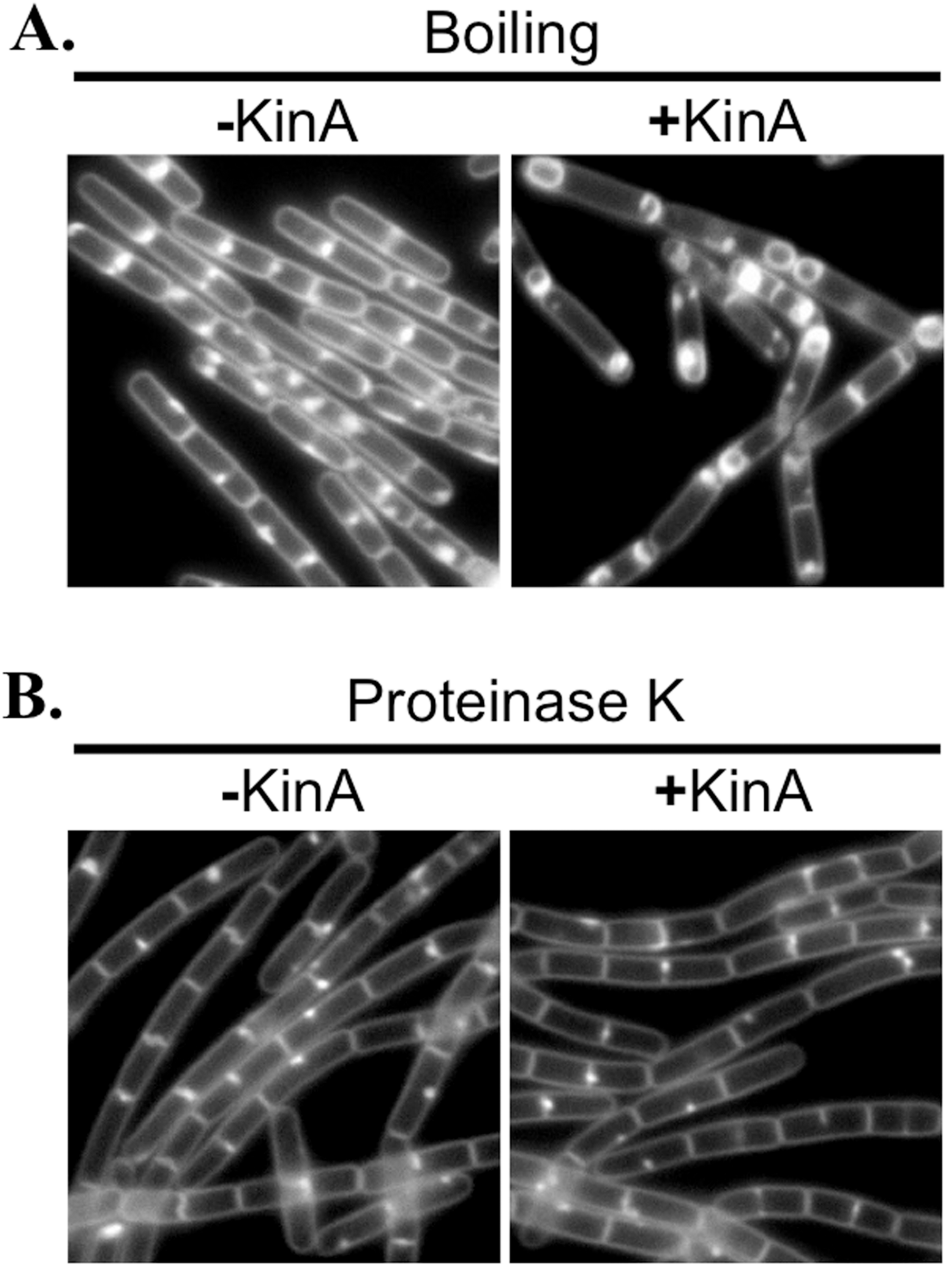
FacX is resistant to boiling, but sensitive to Proteinase K. (A) Conditioned media was boiled for 15 min and utilized in the sporulation assay (Fig 2A) with strain MF1913 (P_*hy-spank*_-*kinA*). (B) Conditioned media was treated with proteinase K and utilized in the sporulation assay with strain MF1913 (P_*hy-spank*_-*kinA*). When indicated, KinA was induced with IPTG (20 μM). Cells were grown for 2 hrs at 37°C before image capture. Membranes were stained with TMA.

Since the characterized quorum-sensing signals of *B*. *subtilis* are peptides, we tested the susceptibility of FacX to heat and proteinase K digestion. FacX’s sporulation stimulating activity was mildly affected by boiling (Fig 4A), but the activity was completely lost following proteinase K digestion (Fig 4A). These experiments were repeated on three independent cultures with three independent batches of conditioned media, each time with similar results. These results indicate that FacX is somewhat heat stable and composed, at least in part, of protein.

**Fig 5:**
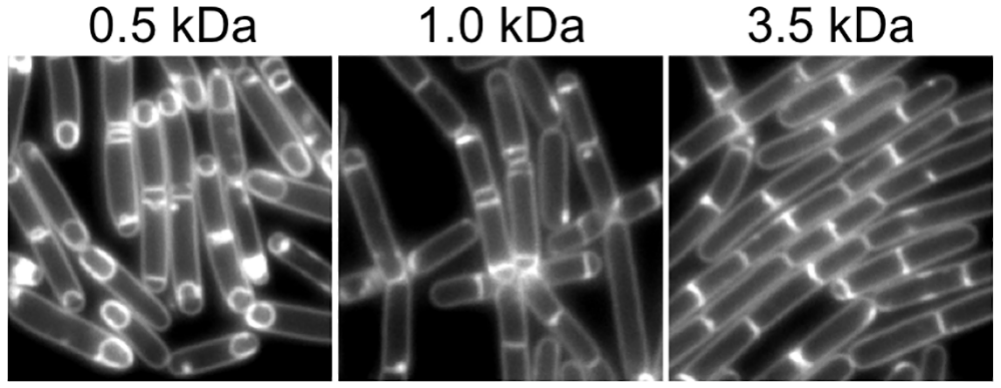
FacX activity is dialyzable. Conditioned CH medium was dialyzed against fresh CH using dialysis tubing with the indicated molecular weight cutoff. The dialysate was then used to perform the KinA-dependent sporulation assay as shown in Fig 2A with strain MF1913. When indicated, *kinA* expression was induced with IPTG (20 μM). Cells were grown for 2 hrs at 37°C before image capture. Membranes were stained with TMA.

To determine the approximate size of FacX, we assayed for KinA-dependent sporulation at low cell densities using dialyzed conditioned media. We found that FacX activity, as judged by our microscopy-based assay, was fully retained by a 0.5 kDa cutoff dialysis tubing (Fig 5). Some FacX activity was lost following incubation with a 1 kDa tubing (Fig 5); the morphological signs of sporulation lagged approximately 20 min behind, but was still robust. In contrast, dialysate from 3.5 kDa cutoff tubing appeared more similar to the uninduced control (Fig 5), suggesting that FacX is likely smaller than 3.5 kDa. These experiments were repeated on three independent biological replicates with three independent batches of conditioned media with similar results. We conclude that at least one component of FacX activity is between 500 and 3,500 Daltons, but more likely between 500 and 1,000 Daltons in size. We do not exclude the possibility that more than one molecule is responsible for FacX activity.

### FacX activity is not dependent on SigH, Spo0A, ComX, or the Opp and App peptide transporters

**Fig 6:**
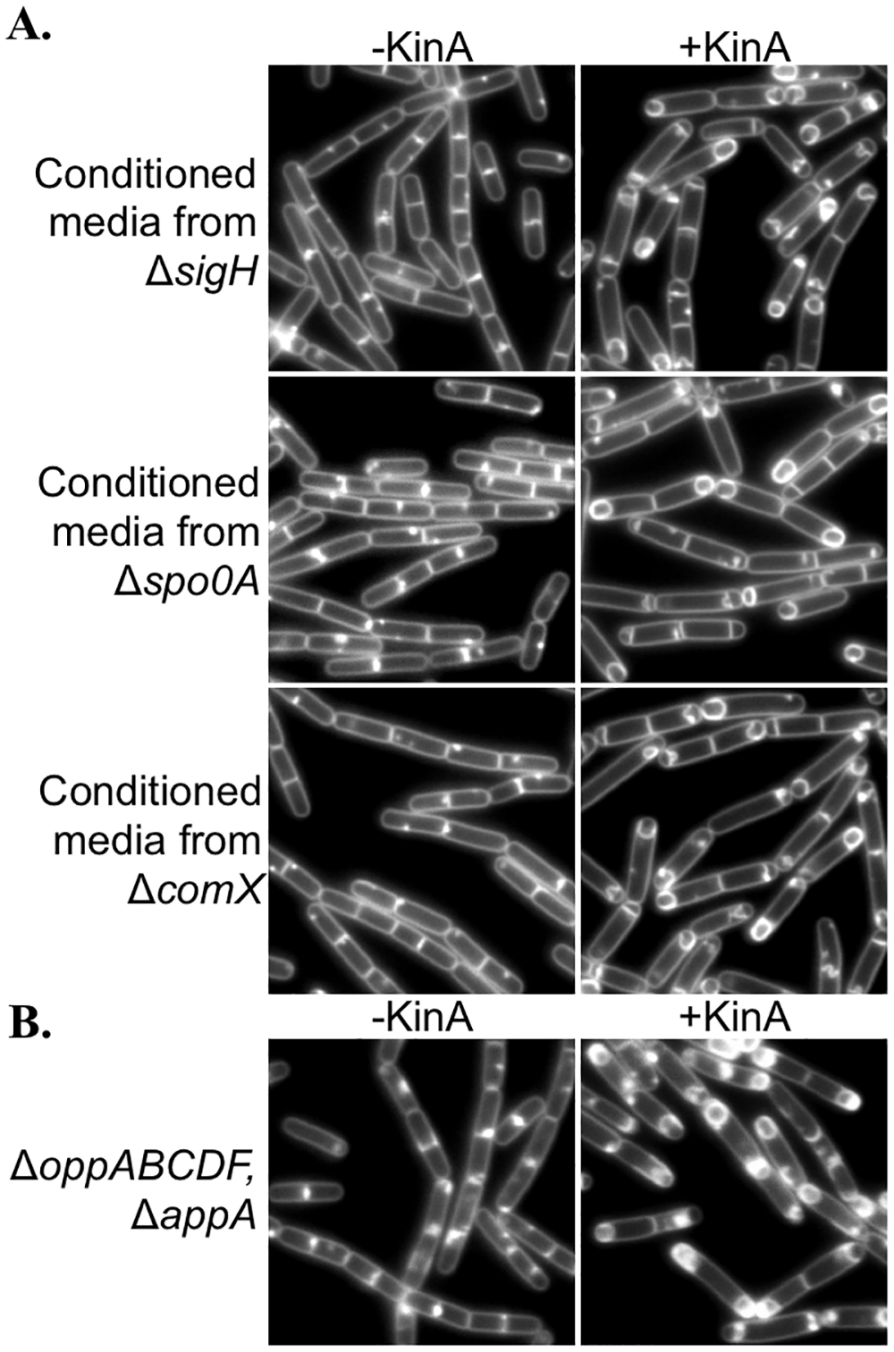
FacX activity is not dependent on Spo0A, SigH, or ComX for production, and does not require the Opp or App peptide uptake systems. (A) A strain harboring P_*hy-spank*_-*kinA* (MF1913) was grown 2 hrs in the indicated media at 37°C before image capture. (B) A strain containing P_*hy-spank*_-*kinA* in an Δ*oppABCDF*, Δ*appA* double mutant (BQA121) was grown 2 hrs at 37°C before image capture. The conditioned media utilized in this experiment was obtained from PY79. When indicated, KinA expression was induced with IPTG (20 μM). Membranes were stained with TMA.

The secreted peptides produced from PhrA, PhrC, and PhrE fall within the size range of interest (500–3,500 Daltons) that are known to contribute to sporulation [32,40,41]. *phrC* and *phrE*, fall within the SigH regulon, and are maximally expressed as *B*. *subtilis* exits exponential growth [30]. To determine if FacX was a previously described sporulation modulating peptide or factor, and to better understand the requirements for FacX synthesis, we performed three sets of experiments. First, we prepared conditioned media from cells lacking SigH or Spo0A, which regulate the expression of a number of post-exponential phase gene products [20,25,27,42]. Conditioned media collected from either Δ*sigH* or Δ*spo0A* mutant cultures were still capable of stimulating sporulation at low cell densities when combined with KinA induction (Fig 6). 95% of cells grown in Δ*sigH* conditioned media (n = 437) and 74% of cells grown in Δ*spo0A* conditioned media (n = 394) displayed polar septa or forespores. In comparison, for the uninduced controls less than 1% of cells grown in Δ*sigH* conditioned media (n = 626) or Δ*spo0A* conditioned media (n = 406) displayed polar septa or forespores. These experiments were repeated with three independent biological replicates in media obtained from three independent batches of conditioned media with similar results. These results indicate that FacX does not depend on SigH or Spo0A to accumulate to sporulation-inducing levels.

Next, we investigated the possibility that the oligopeptide pheromone ComX, which accumulates in post-exponential phase media and is of comparable size to FacX [1], might account for the FacX activity we observed. To test this possibility, we obtained conditioned media from a Δ*comX* strain and repeated the KinA-dependent sporulation assay. As shown in Fig 6A, cells were still capable of sporulating via KinA induction in conditioned media obtained from a Δ*comX* strain. 86% of cells grown in Δ*comX* conditioned media (n = 436) displayed polar septation or forespores, compared to less than 1% (n = 421) in the uninduced control. Similar results were obtained with two independent biological replicates. These results indicate that ComX is not the factor present in conditioned media that permits sporulation initiation via artificial KinA expression.

The oligopeptide signals involved in regulating *B*. *subtilis* sporulation (PhrA, CSF, and PhrE) are known to be transported back into *B*. *subtilis* through the Opp [31,32,40] and App [33] peptide uptake systems, so we tested if FacX requires the Opp or App transporters for its activity. To create a strain null for Opp and App activity, we generated a double mutant in which an essential component of the *app* operon, *appA [33]* and the entire Δ*oppABCDF* operon w deleted. We then assayed for KinA-dependent sporulation at low cell densities in the Δ*oppABCDF*, Δ*appA* double mutant background. The cells initiated sporulation efficiently in conditioned media following KinA induction (Fig 6), similar to the wildtype control (Fig 2B). 91% of the Δ*oppABCDF*, Δ*appA* mutant cells (n = 345) displayed polar septa or forespores when grown in conditioned media collected from PY79, compared to 2% of uninduced control cells (n = 265). This experiment was repeated with two independent biological replicates with similar results. Since FacX does not require SigH, Spo0A, or ComX for synthesis, and does not require Opp or App for transport, our results suggest that FacX constitutes one or more molecules not previously shown to promote sporulation.

### FacX creates a permissive condition for sporulation

**Fig 7:**
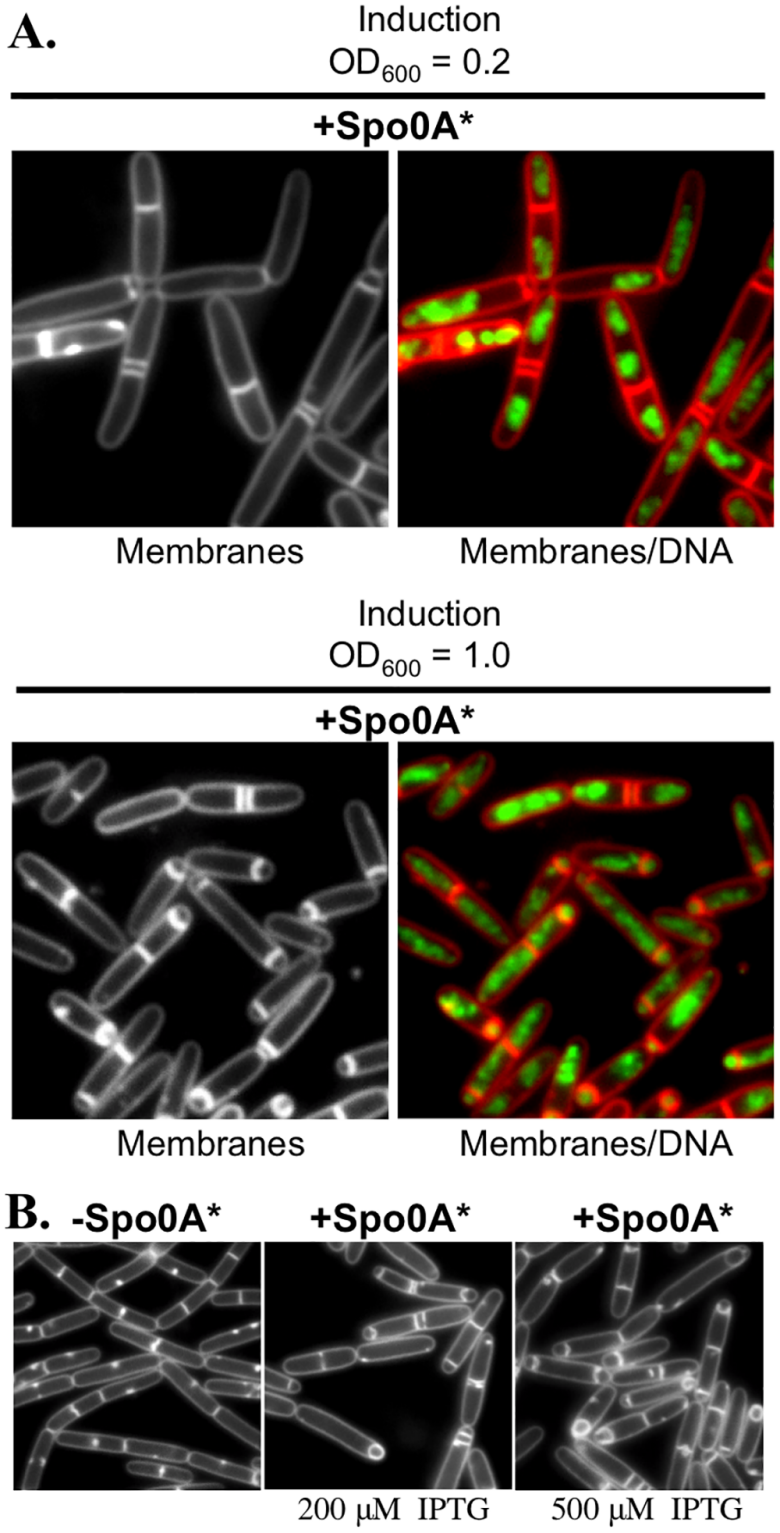
*Bacillus* can be induced to sporulate through expression of a constitutively active allele of Spo0A (Spo0A*) if induced during stationary phase or grown in conditioned media. (A) A strain harboring P_hy-spank—_ Spo0A* (MF2146) was grown in CH at 37°C, induced by the addition of IPTG (200 μM) at the indicated optical densities, and grown for 2 hrs at 37°C before image capture. Membranes (white and red) were stained with FM4-64, and DNA (green) was stained with DAPI. (B) A strain harboring P_*hy-spank*_-Spo0A* (MF2146) was grown in conditioned media obtained from PY79 as shown in Fig 2A. When indicated, Spo0A* was induced by the addition of IPTG (either 200 μM or 500 μM, as indicated, beginning OD_600_ 0.04) and grown for 2 hrs at 37°C before image capture. Membranes were stained with TMA.

The observation that KinA-dependent sporulation at low cell densities required FacX lead us to revisit a study which concluded that sporulation requires a gradual accumulation of Spo0A-P [29]. In this study, the authors proposed that the acute induction of Spo0A* (the constitutively active allele of Spo0A) was unfavorable for sporulation since it also led to the acute expression (and repression) of both low and high threshold Spo0A-regulated genes. In light of our new findings, we hypothesized that sporulation through artificial KinA induction requires two factors: the accumulation of sufficient levels of active Spo0A (acute or gradual) and FacX. If this hypothesis is correct, then the induction of Spo0A* in cells growing in the presence of FacX should be sufficient to allow sporulation. To test this idea, we first inoculated exponentially growing cells harboring P_*hy-spank*_-Spo0A* at a low optical density. These conditions were previously shown to induce Spo0A-regulated genes [20], but not result in sporulation [29]. Consistent with prior findings, we found that Spo0A* induction at low optical densities did not result in sporulation. Instead, cells exhibited pleotropic phenotypes consistent with the up-regulation of high-threshold Spo0A-P genes [20], including asymmetric septa (6% of n = 366 cells) [43] and movement of chromosomes towards poles [44]. The cells also appeared unhealthy, frequently lysed, and did not progress in sporulation, as judged by the lack of engulfing forespores. In contrast, when Spo0A* was induced during post-exponential phase (induction at OD_600_ values of 1.0–1.5) sporulation phenotypes were observed, including engulfing forespores (Fig 7A). Sixty percent of cells (n = 699) displayed polar septa or forespores following 2 hr induction with 200 μM IPTG, compared to less than 1% of uninduced cells (n = 595). Similarly, cells expressing Spo0A* could also be induced to initiate sporulation at low cell densities when they were grown in conditioned media (Fig 7B). Forty percent of cells harboring P_*hy-spank*_-spo0A* (n = 699) exhibited polar septation or forespores following the addition of 200 μM IPTG. When the same strain was induced with 500 μM IPTG, 58% (n = 586) of cells possessed polar septa or forespores. Similar results were obtained in four independent biological replicates. These results suggest that expression of Spo0A* can support the initiation of sporulation if a second signal, such as FacX, is also present.

## Conclusions

We report that cultures of *B*. *subtilis* secrete a novel extracellular factor or factors, FacX, that accumulates with cell density in CH medium. FacX is highly stable and exhibits many of the characteristics of previously characterized peptide-based signaling molecules of *B*. *subtilis*, including susceptibility to proteases and relatively small size (less than 3.5 kDa). However, our data argue against the possibility that FacX is a previously described peptide for several reasons. First, FacX does not require the Opp and App transport systems for its activity (Fig 6B). In contrast, the Phr peptides previously shown to have a role in sporulation (PhrA, CSF, and PhrE) require the Opp ABC transporter for activity [17]. Of note, the *opp* system is required for efficient sporulation and is reduced in sporulation efficiency compared to wildtype [31], but we find the cells can be induced to initiate sporulation with similar efficiencies to wildtype (as judged by polar septation and forespore counts) when KinA is artificially expressed. Presumably this bypass of wildtype phosphorelay regulation results from the artificial KinA expression shifting Spo0A towards its phosphorylated form. The bypass of Phr-dependent signaling in the P_*hy-spank*_-*kinA* strain is also supported by the observation that FacX activity is not appreciably affected in the absence of SigH. Most *phr* genes have SigH-dependent promoters [25,30]; expression of *phrC* is undetectable in a *sigH* mutant [45] and 50% lower in a *phrE* mutant [41], yet we do not observe major differences in sporulation initiation in the SigH mutant when it is induced to sporulate via artificial KinA expression (Fig 6A). We also found that conditioned media obtained from a Δ*comX* strain supports sporulation at low cell densities via artificial KinA induction (Fig 6A). Thus, even though the ComX pheromone accumulates with cell density and is similar in size [1], it is not required to stimulate sporulation at low cell densities via artificial KinA induction.

FacX allows cells growing at low optical densities to initiate sporulation following induction of Spo0A* (Fig 7). These results suggest that FacX either creates or signals a permissive condition for sporulation. The induction of Spo0A* in CH media at low optical densities (Fig 7A) shows that exponential growth and the induction of active Spo0A are not sufficient to induce efficient sporulation, consistent with prior findings [26,29]. However, we find that when cells are grown in conditioned media containing FacX, sporulation initiation via Spo0A* induction is possible, even at low cell densities (Fig 7). This suggests that FacX is unlikely to act through the phosphorelay that regulates Spo0A-P accumulation, as the Spo0A* variant works independently of phosphorylation.

We also show that KinA induction is not sufficient to induce sporulation at low cell densities during exponential growth. Instead, we find that sporulation via artificial induction of KinA also requires exit from exponential phase (Fig 1). These results are in seeming contradiction to the findings of Fujita and Losick, who reported that artificial induction of KinA at low cell densities (OD_600_ of 0.05 in CH), resulted in expression from an early sporulation promoter, P_*spoIIG*_, suggestive of entry into sporulation [29]. This apparent contradiction can be explained by the fact that the authors of the prior study only visually assayed for sporulation in populations that had exited post-exponential phase. Consistent with this idea, we also found that post-exponential phase growth is permissive for KinA-dependent sporulation (Fig 1).

We observe that FacX activity only reaches sufficient levels to promote efficient sporulation in cultures that are exiting exponential phase. However, this does not necessarily mean that FacX production is growth phase dependent. It is also possible that the FacX is expressed constitutively, and only accumulates to sufficient levels as cells achieve post-exponential phase densities. Rapidly falling GTP levels (caused by the addition of the drug decoyinine [16] can also create a condition permissive for sporulation at low cell densities when Spo0A* is induced. We speculate based on this observation, and the fact that FacX does not appear to act through the phosophorelay regulating Spo0A-P accumulation, that FacX may either cause or signal falling GTP levels in the cell. Similar to decoyinine addition, this could promote a permissive state for sporulation. Regardless, of FacX’s identity and mechanism-of-action, our results are consistent with the conclusions of Ireton et al. over two decades ago [26]; the ultimate decision of whether or not to sporulate requires not only sufficient levels of active Spo0A, but also at least one other signal or condition.

Our attempts to determine the molecular identity of FacX from conditioned media have thus far been unsuccessful. However, we identified several properties of FacX activity that may be informative. For example, FacX activity was not bound by either anionic or cationic exchange resins, suggesting it may have a non-polar character. Consistent with this idea, FacX binds efficiently to C18 resin, but conditions to elute the activity from the column in a peak fraction were not successful. These results may also suggest that FacX activity is comprised of more than one molecule or requires additional processing to a mature form before it is active. Future experiments will be aimed at determining the identity of FacX and investigating how FacX changes the physiology of the cells, with the ultimate goal of better understanding how bacteria integrate environmental cues with complex developmental decisions like sporulation.
